# Association of circular RNAs and environmental risk factors with coronary heart disease

**DOI:** 10.1186/s12872-019-1191-3

**Published:** 2019-10-16

**Authors:** Yi Sun, Rong Chen, Shaowei Lin, Xiaoxu Xie, Hailing Ye, Fuli Zheng, Jie Lin, Qing Huang, Shuna Huang, Qishuang Ruan, Tingxing Zhang, Huangyuan Li, Siying Wu

**Affiliations:** 1 Department of Epidemiology and Health Statistics, School of Public Health, Fujian Medical University, Minhou County, Fuzhou, Fujian China; 2 National Research Institute for Health and Family Planning, Beijing, China; 3 Department of Preventive Medicine, School of Public Health, Fujian Medical University, Minhou County, Fuzhou, Fujian China; 4 Department of Orthopedics, Fujian Medical University Union Hospital, Fuzhou, Fujian China; 5 Department of Cardiology, The First Affiliated Hospital of Fujian Medical University, Fuzhou, Fujian China

**Keywords:** Coronary heart disease, Circular RNA, Environmental risk factors, Microarray, Interaction

## Abstract

**Background:**

Coronary heart disease (CHD) is a complex disease caused by multi-factors and a major threat to human health. Circular RNAs (circRNAs) have critical roles in various biological processes and diseases. This study explores the independent role of circRNAs and their interaction with environmental factors in CHD.

**Methods:**

A case–control study was conducted from March 2015 to September 2017 in Fuzhou, China. A total of 585 CHD patients and 585 gender- and age-matched healthy controls were enrolled. Questionnaire survey, health examination and molecular biology laboratory testing were conducted. Microarray technology and quantitative real-time polymerase chain reaction (PCR) were used to profile the expression levels of circRNAs. The area under the curve (AUC) of the receiver operating characteristic (ROC) was used to determine the diagnostic cut-offs. Multivariate logistic regression and multiplicative analysis were used to analyse the effects of environmental factors and hsa_circ_0008507, hsa_circ_0001946, hsa_circ_0000284 and hsa_circ_0125589 on CHD.

**Results:**

The expression profile of circRNAs showed that 3423 circRNAs were differentially expressed at *P* < 0.05, but none pass multiple testing correction. qRT-PCR further confirmed the expression levels of hsa_circ_0008507, hsa_circ_0001946 and hsa_circ_0000284 in peripheral blood leukocytes in CHD cases were higher than those in non-CHD subjects (All *p* < 0.05). Hsa_circ_0008507 (OR = 1.29; 95% CI: 1.11–1.50), hsa_circ_0001946 (OR = 1.20; 95% CI: 1.01–1.42) and hsa_circ_0000284 (OR = 2.05; 95% CI: 1.32–3.19) were independent risk factors for CHD after controlling other common environmental risk factors. The AUC for hsa_circ_0008507, hsa_circ_0001946 and hsa_circ_0000284 was 0.75, 0.71 and 0.68, respectively. Compared with non-smoking individuals with low hsa_circ_0008507 expression, the smokers with high hsa_circ_0008507 expression showed the highest magnitude of OR in CHD risk. Additionally, a statistically significant multiplicative interaction was found between hsa_circ_0008507 and smoking for CHD.

**Conclusions:**

Hsa_circ_0008507, hsa_circ_0001946 and hsa_circ_0000284 were closely related to the occurrence and development of CHD. The combination of smoking and high hsa_circ_0008507 expression causes the occurrence and development of CHD.

**Electronic supplementary material:**

The online version of this article (10.1186/s12872-019-1191-3) contains supplementary material, which is available to authorized users.

## Background

Coronary heart disease (CHD) is a leading global cause of death with annually increasing mortality and morbidity [1, 2]. Family history of CHD, heavy drinking, arterial hypertension, smoking, obesity, diabetes mellitus, hypertension and physical activity are classic risk factors for CHD [3–5]. CHD is also a polygenic hereditary disease [6]. Epigenetics are called “bridges” that connect environmental factors and the genomes affecting gene expression in the absence of genomic variation, thereby affecting the sensitivity of the disease [7–9].

Circular RNAs (circRNAs) are currently noticeable in the field of RNA. They form covalently fused loops, where the RNA’s 5′ end fuses to its 3′ end and removes the 5′ Cap and poly(A) tail [10]. Recent studies suggested that circRNAs are widespread, abundant, conserved and tissue-specific endogenous ncRNAs in mammalian cells [11, 12]. CircRNAs regulate gene expression by acting as miRNA sponges, RNA-binding protein sequestering agents or nuclear transcriptional regulators [13, 14]. Despite the important role of circRNAs in the development of CHD, their relative contributions and interaction with environmental factors to CHD remain largely unknown.

In this study, 585 CHD patients and 585 healthy controls were enrolled to evaluate the effects of environmental factors on CHD risk. Microarray and experiments involving these populations were conducted to explore the roles of dysregulated circRNAs in the CHD cases. circRNA have been tested in tissues, serum, exosomes and other body fluids in various diseases [15, 16]. In this study, we also validated the differentially expressed circRNAs in whole blood and peripheral blood leukocyte samples to further clarify our results. This study aims to explore the independent role of circRNAs and their interaction effect with environmental factors in the CHD.

## Methods

### Study population

A frequency-matched case-control study was conducted in which case groups and controls must have the same gender and age ratio (±3 years). A research factor with a small OR was selected to calculate the appropriate sample size, according to the pre-experimental results and considered the following: α = 0.05, β = 0.10, P_0_ = 0.260 and OR = 1.70. Calculations using PASS revealed N_1_ = N_2_ = 379 people. In this study, 1300 questionnaires, including 650 cases and 650 controls, were distributed, and all were collected between March 2016 and September 2017 from the First Affiliated Hospital of Fujian Medical University and the Affiliated Union Hospital of Fujian Medical University, China. After corresponding the age and gender, 585 CHD patients and 585 controls were enrolled in this study. CHD was defined using the following criteria: (1) significant stenosis (≥ 50%) of > 1 major coronary artery was confirmed by present cardiac catheterisation, (2) documented history of prior myocardial infarction (MI) and a prior coronary revascularisation procedure (percutaneous coronary intervention or coronary artery bypass graft), (3) patients in the stable stage after acute MI and patients with ST-segment elevation/depression on ECG. The subjects without medical history of cardiovascular diseases were selected as the non-CHD subjects. All subjects were long-term residents in Fujian, subjects with other types of heart disease, serious brain organic diseases, malignant tumours, hepatic or renal dysfunction, recent infections and endocrine system diseases were excluded from the study. This study protocol conformed to the ethical guidelines of the Declaration of Helsinki. The protocol was approved by the ethics committee of Fujian Medical University School. All selected patients and controls provided informed consent.

### Questionnaire survey

All interviewer who conducted the standard questionnaire were specially trained. The questionnaire included demographic characteristics (age, gender, marital status, educational level), lifestyle habits (smoking, alcohol drinking, diet, exercise), social psychological factors (, anxiety and depression levels), Physiological index (height, weight, waist circumference (WC)) and family history of cardiovascular disease. The questionnaire was listed in Additional file 1: Table S1. Individuals who have consumed more than 20 packs of cigarettes or one cigarette a day for at least 1 year were defined as smokers. Alcohol drinkers were defined according to literature [17]. Each exercise lasting for no less than 20 min will be recorded as an effective physical exercise, according to this standard, the weekly physical activity of all subjects were determined. Anxiety and depression were evaluated by self-rating depression scale (SDS) and self-rating anxiety scale (SAS) [18]. SAS scores were divided into < 50, 50-59, 60-69, and ≥ 70, which were used to indicate normal, mild anxiety, moderate anxiety, and severe anxiety, respectively. SDS was categorised as < 53, 53–62, 63–73 and ≥ 74, which were used to indicate normal, mild depression, moderate depression, and severe depression, respectively. Body mass index (BMI) = weight (kg) / [height (m)]^2^ and categorised to three scales as follows: < 18.5 kg/m^2^ underweight, 18.5–24.0 kg/m^2^ normal and ≥ 24.0 kg/m^2^ overweight and obese. A male waistline cutoff of 85 or higher means abdominal obesity, and a female waistline cutoff of 80 or more means abdominal obesity. According to gender, WC was classified into two categories: A male WC with a cutoff of 85 or higher implies abdominal obesity, whereas a female WC with a cut-off value of 80 or more suggests abdominal obesity.

### CircRNA microarray analysis

The samples used for microarray analysis include 4–12 cases [19]. Therefore, five patients with similar age, disease and disease duration and with no other diseases and five controls with similar general conditions, age and sex was selected for microarray analysis. The distribution of demographic variables is listed in Additional file 2: Table S2. The RNAs of the peripheral blood samples from five CHD cases and five non-CHD subjects were extracted for microarray analysis. According to the manufacturer’s instructions, total RNA was extracted from 1 mL of whole blood sample by using a fast total RNA extraction kit (Bioteke, Beijing, China). RNA was then dissolved in RNase-free water. NanoDrop1000 instrument (NanoDrop, England) was used to measure the yield and purity of the RNA.. All total RNA was of high purity with OD260 / 280 in the range of 1.80-2.00. 1% formaldehyde denaturing gel electrophoresis was used to determine the integrity of the RNA. The extracted RNAs were digested, dephosphorylated, denatured, amplified and labelled with Cy3-dCTP according to the manufacturer’s specifications. The purified RNAs were hybridised to a microarray (Agilent human circRNA Array V2.0) containing 170,340 human circRNA probes. GeneSpring software V13.0 (Agilent Technologies, Santa Clara, CA, USA) was then used to analyze microarray data for circRNA. The thresholds were fold change (FC) of > 2 or < − 2 and *p* < 0.05 according to the t-test. The data were log-2 transformed and median-centred by genes through CLUSTER 3.0 software and then analysed using hierarchical clustering with average linkage.

### RNA extraction

Representative subjects were selected using stratified sampling from the first part of the study. The subjects in the case group were divided into two layers according to age (i.e. < 65 years old and ≥ 65 years old) and stratified by gender according to each age group. The sex ratio of all subjects in the case group were determined. According to the random number table, the corresponding number of male and female subjects was finally selected at each age level. The control group were also sampled using the same method. According to the pre-experimental results, the PASS software obtained the following result: N_1_ = N_2_ = 25, considering α = 0.05, β = 0.10, P_0_ = 0.50 and OR = 1.8. Therefore, at least 25 samples were required in each group. Therefore, 30 cases and controls were finally determined for peripheral blood quantitative real-time polymerase chain reaction (qRT-PCR) validation, and 100 cases and controls were used for peripheral blood leukocyte qRT-PCR validation. A fast total RNA extraction kit (Bioteke, Beijing, China) was used to extract total RNA from peripheral blood in 30 CHD patients and 30 non-CHD controls. Total RNA from peripheral blood leukocytes in 100 CHD patients and 100 non-CHD controls was extracted by using TRIzol reagent (Invitrogen, USA). The examination of RNA concentration, purity, and integrity were as described above.

### cDNA synthesis and qRT-PCR

Reverse transcription of quantified RNA was performed using PrimeScript RT Reagent Kit (Takara Bio Inc., Shiga, Japan) according to the manufacturer’s instructions. qRT-PCR was used to measure the expression levels of circRNAs, Which was performed on the LightCycler 480 Real-Time PCR System (Roche, Switzerland) with the SYBR® Premix Ex Taq™ II kit (Takara Bio Inc., Shiga, Japan). The reaction conditions were listed as follows: Amplification curves were obtained by 45 cycles of 95 °C 30 s, 95 °C 5 s, 60 °C 34 s, whereas dissolution curves were obtained by one cycle of 95 °C 15 s, 60 °C 1 min, 95 °C 15 s. GAPDH was used as internal control. 2^-△△CT^ method was used to calculate the expression levels of circRNAs. The primer sequences are shown in Additional file 3: Table S3.

### Statistical analyses

Data with normal distribution were presented as the mean value±SD and compared using two-tailed Student’s t-test. Skewed data were represented as median (25th–75th quartile) and compared using Mann–Whitney U test. Discrete variables were displayed as percentages, distribution differences were examined by the chi-square (χ^2^) test. The diagnostic cut-offs of circRNAs were obtained from the receiver operating characteristic (ROC) curve. Univariate analysis of meaningful variables was included in the multivariate analysis. Crossover analysis was used to assess the association between CHD and risk factors. A *p*-value < 0.05 (two-tailed) was considered significant. All statistical analyses were performed using SPSS 25.0 software.

## Results

### Baseline demographic characteristics

The distribution of all subjects according to demographic variables is shown in Table 1. No significant difference was found in their general demographic characteristics, including age, gender, marital status and educational level (all *p* > 0.05), thereby indicating that the frequency matching was adequate (Table 1).

**Table 1:** Baseline characteristics of study subjects

Characteristics	CHD, n(%)	Non-CHD, n(%)	χ^2^	*p*-value
Gender			0.000	1.000
Male	318 (54.4)	318 (54.4)		
Female	267 (45.6)	267 (45.6)		
Age			0.777	0.378
< 65	271 (46.3)	256 (43.8)		
≥ 65	314 (53.7)	329 (56.2)		
Marital status			0.380	0.538
Marriage	536 (91.6)	530 (90.6)		
Single and others	49 (8.4)	55 (9.4)		
Education level			1.108	0.575
Below primary school	260 (44.4)	263 (45.0)		
Middle school	257 (43.9)	265 (45.3)		
College or higher	68 (11.6)	57 (9.7)		

*CHD* coronary heart disease, *Non-CHD* None coronary heart disease

### Environmental factors and CHD

Univariate analysis on the associations between individual behavioural factors and risk of CHD showed that salty diet. Social psychological factor analysis showed that anxious people are highly prone to CHD. BMI (≥24.00) and abdominal obesity were significantly different in CHD and non-CHD subjects. However, light diets and physical exercise (one to two times per week) were protected factors for CHD. Unconditional logistic regression analysis was further used to evaluate the associations between the environmental factors (high-salt diet, light diet, active exercise, anxiety, BMI and abdominal obesity) and CHD. Significant increased risk effects for CHD were associated with anxiety {odds ratio (OR) = 2.34; 95% confidence interval (95% CI): 1.67–3.28} and being overweight (BMI ≥ 24.00) {OR = 1.47; 95% CI: 1.13–1.91}. Physical exercise (one to two times per week) {OR = 0.41; 95% CI: 0.23–0.75} was the protective factor for CHD (Table 2).

**Table 2:** Univariate and Multiple logistic regression analysis of environmental factors and CHD risk

Factors	Univariate logistic regression		Multivariate logistic regression	
	OR (95% CI)	*p*-value	OR^a^ (95% CI)	*p*-value
Smoking	1.06 (0.83-1.35)	0.661	–	–
Passive smoking	1.08 (0.82-1.43)	0.569	–	–
Alcohol drinking	1.06 (0.76-1.46)	0.741	–	–
High-salt diets	1.75 (1.35-2.28)	< 0.001	1.20 (0.76-1.88)	0.438
Light diets	0.60 (0.47-0.76)	< 0.001	0.78 (0.51-1.20)	0.260
Physical exercise				
< 1 time/week	1.00	–	1.00	–
1-2 times/week	0.45 (0.25-0.80)	0.007	0.41 (0.23-0.75)	0.004
≥ 3 times/week	0.96 (0.75-1.22)	0.713	0.94 (0.73-1.21)	0.608
Character				
B type	1.00	–	1.00	–
A type	1.29 (0.99-1.69)	0.060	–	–
C type	1.43 (0.93-2.17)	0.105	–	–
D type	1.56 (0.68-3.55)	0.294	–	–
Anxiety	2.32 (1.69-3.19)	< 0.001	2.34 (1.67-3.28)	< 0.001
Depression				
Normal	1.00	–	1.00	–
Mild	1.33 (0.93-1.90)	0.120	–	–
Moderate/Severe	1.40 (0.84-2.35)	0.196	–	–
BMI				
18.50–23.99	1.00	–	1.00	–
< 18.50	0.69 (0.39-1.21)	0.192	0.68 (0.37-1.22)	0.194
≥ 24.00	1.50 (1.18-1.90)	0.001	1.47 (1.13-1.91)	0.004
Abdominal obesity	1.41 (1.08-1.86)	0.013	1.11 (0.81-1.51)	0.530
Family history of hypertension	1.16 (0.91-1.47)	0.228	–	–
Family history of stroke	1.44 (0.92-2.25)	0.114	–	–
Family history of diabetes	1.14 (0.82-1.59)	0.445	–	–

*OR* odds ratio, *CI* confidence interval

^a^ Data are adjusted for age, gender, marital status, and education level, Statistically significant variables in univariate analysis were selected for further multivariate analysis

### Identification of dysregulated circRNA expression profiles

**Fig. 1:**
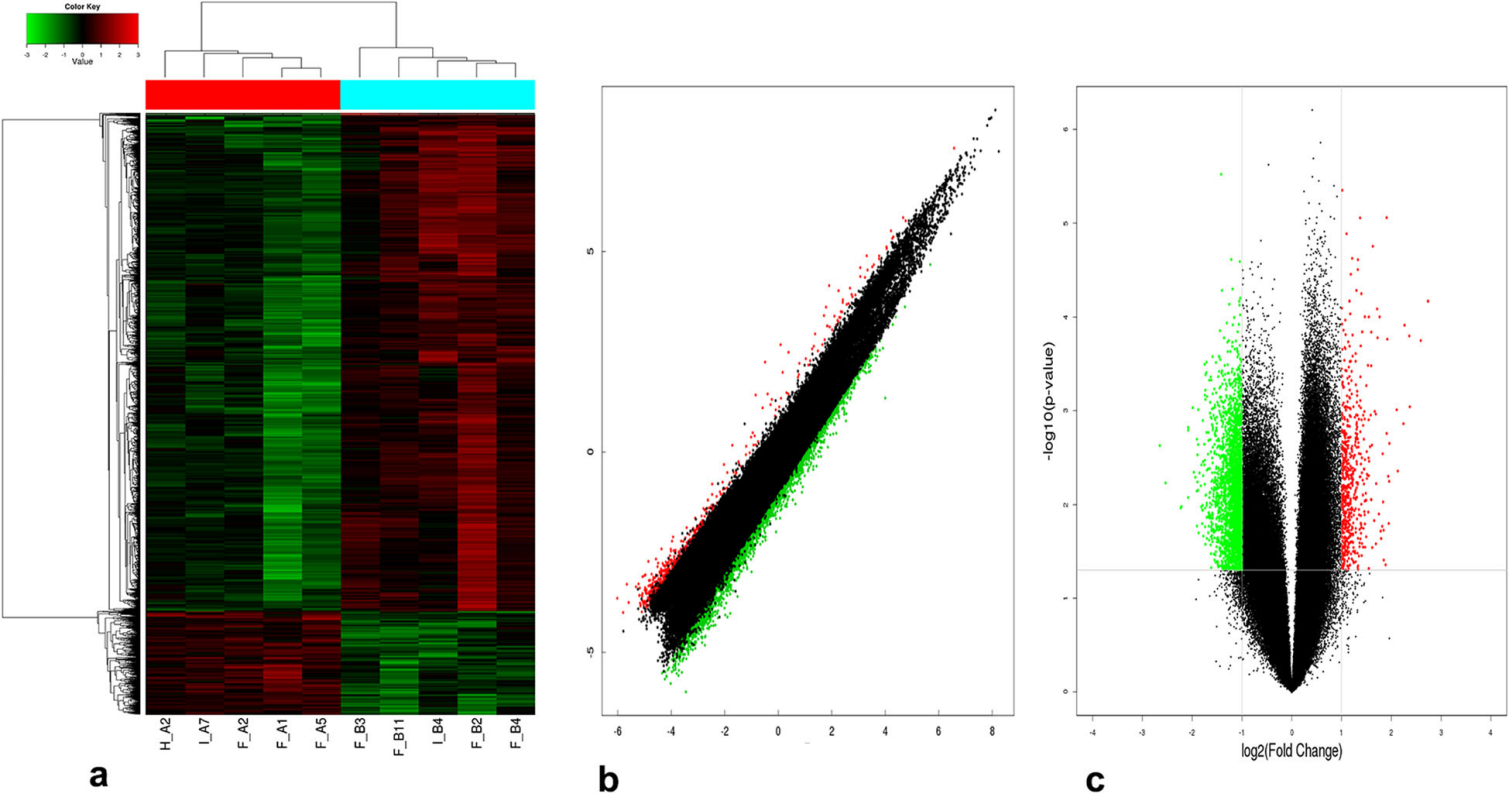
Differentially expressed circRNAs in CHD cases versus non-CHD subjects: **a** Heat map. Heat map analysis from 10 samples in the CHD cases (F_A2, F_A1, F_A5, H_A2 and I_A7) and non-CHD subjects (F_B3, F_B11, F_B4, F_B2 and I_B4) revealed different circRNA expression profiles. Red indicates relatively up-regulated circRNAs, green represents relatively down-regulated circRNAs, and black indicates no significant difference. **b** Scatter plot. Red points indicate up-regulated circRNAs with FC > 2.0 in CHD cases, green points indicate down-regulated circRNAs, and black indicates no significant difference. **c** Volcano plot. Red or green points represent up-regulated or down-regulated circRNAs (FC > 2.0, *p* < 0.05), respectively, whereas black indicates no significant difference

The heat map, volcano plot and scatter plot of microarray assay showed abnormal expression of circRNAs in the CHD cases (Fig. 1). The microarray study identified that 3423 circRNAs were differentially expressed in CHD and non-CHD subjects based on FC and *p*-value (FC > 2, *p* < 0.05). Four circRNAs were selected for qRT-PCR validation analysis to independently validate our results and determine the roles of circRNAs in CHD. Selection was based on the following: (1) hsa_circ_0008507, hsa_circ_0125589 and hsa_circ_0000284 are among the most abundant and have significantly differentially expression according to microarray analysis, (2) overexpression of hsa_circ_0001946 in cardiac myocyte cells promotes cell apoptosis [20] and silencing hsa_circ_0000284 can alleviate retinal vascular dysfunction [21]. In summary, the circRNAs associated with the occurrence and development of CHD include hsa_circ_0125589, hsa_circ_0008507, hsa_circ_0001946 and hsa_circ_0000284 (Table 3).

**Table 3:** The basic information of 4 selected circRNAs

CircRNA ID	FC	*p*-value	Regulation
hsa_circ_0125589	4.64	0.010	Down
hsa_circ_0008507	4.31	< 0.001	Up
hsa_circ_0001946	1.39	0.088	Up
hsa_circ_0000284	2.24	0.008	Down

*FC* fold change, *circRNA* Circular RNA

### Expression of circRNAs in peripheral blood

**Fig. 2:**
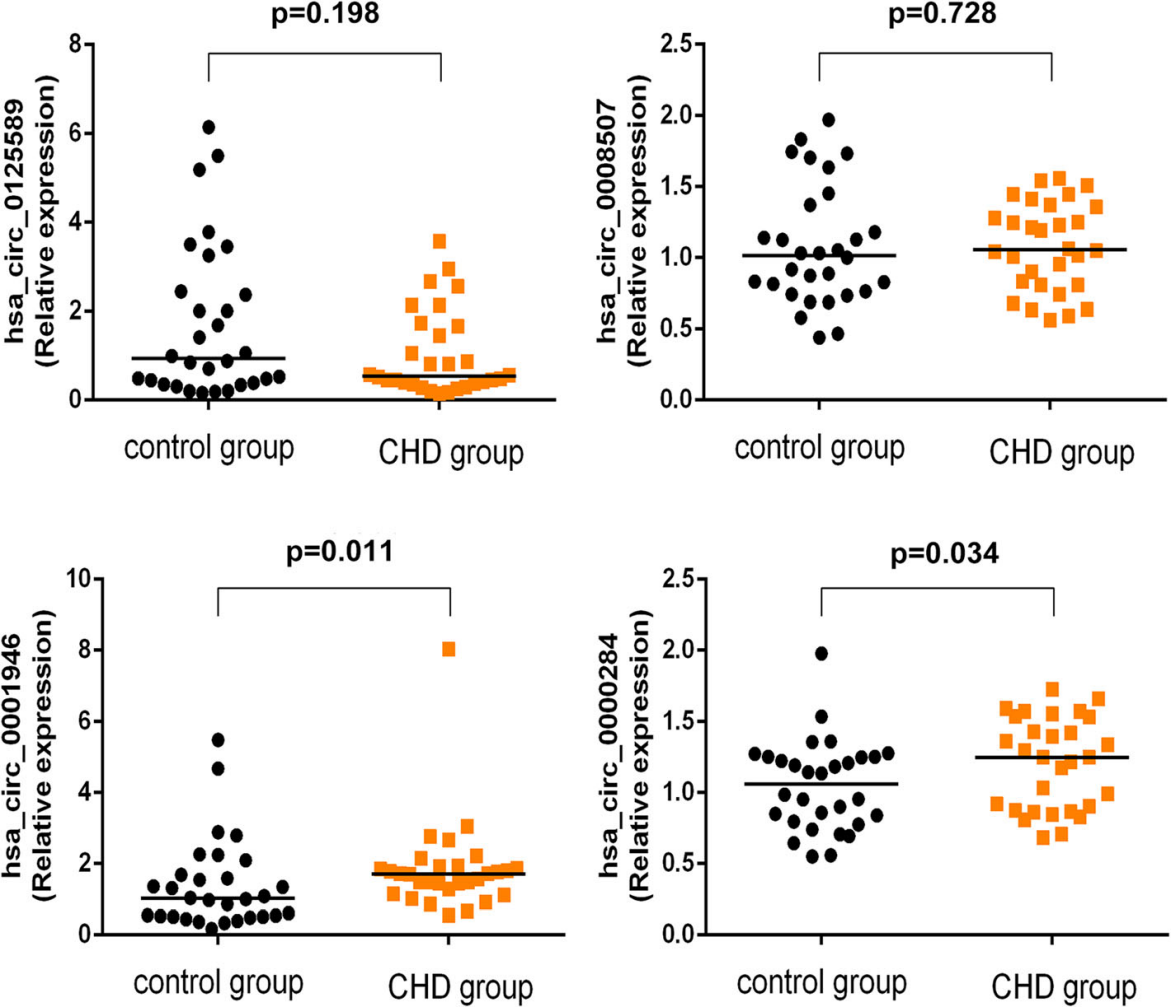
Comparison of the expression levels of circRNAs between CHD and control subjects: four differentially expressed circRNAs were validated by qRT-PCR. Data are expressed as the median (25th–75th quartile)

We selected 30 CHD cases and 30 non-CHD controls with corresponding age and gender as qRT-PCR subjects. The expression levels of hsa_circ_0001946 and hsa_circ_0000284 in CHD were significantly elevated compared with those in the non-CHD subjects (Fig. 2). No significant differences were found in the expression levels of hsa_circ_0125589 and hsa_circ_0008507. The expression level of hsa_circ_0001946 was consistent with the microarray assay, whereas that of hsa_circ_0000284 was contrary to the result of the microarray.

### Validation of dysregulated circRNAs of peripheral blood leukocytes by qRT-PCR

**Fig. 3:**
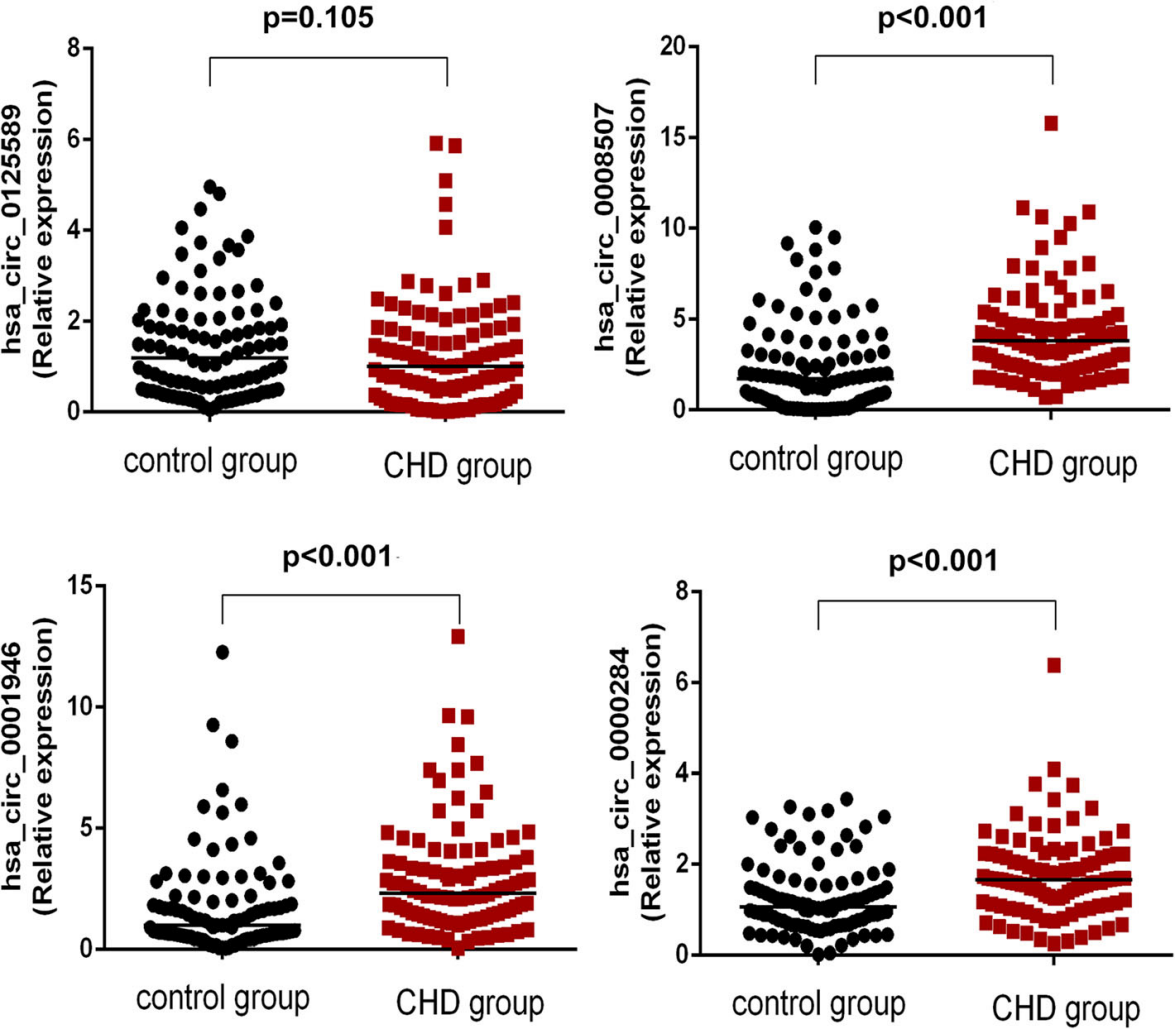
Comparison of the expression levels of circRNAs between CHD and non-CHD subjects of peripheral blood leukocytes: Four differentially expressed circRNAs were validated by qRT-PCR. Data are expressed as the median (25th–75th quartile)

We selected 100 CHD cases and 100 non-CHD controls as qRT-PCR subjects, all of which corresponded in terms of age and gender. The expression levels of hsa_circ_0001946 and hsa_circ_0000284 in the peripheral blood leukocytes in CHD group were significantly elevated compared with those in the non-CHD subjects. No significant difference was found in the expression level of hsa_circ_0125589 in peripheral blood leukocytes between the case and control groups (*p* > 0.05) (Fig. 3). These results were consistent with the qRT-PCR validation of peripheral blood samples. However, the expression level of hsa_circ_0008507 was up-regulated, which was inconsistent with the qRT-PCR validation of peripheral blood samples. The expression levels of hsa_circ_0008507 and hsa_circ_0001946 were consistent with the microarray assay, whereas that of hsa_circ_0000284 was contrary to the result of the microarray assay.

### Multivariate analysis of circRNAs and environmental factors

Two logistic regression analysis models were further used to analyse the association of factors (age, gender, marital status and education level in Model 1 and age, gender, marital status, education level, anxiety, BMI and physical activity in Model 2) with circRNA. CHD was the dependent variable (0 = no, 1 = yes), and the related environmental factors and four verified circRNAs in peripheral blood leukocytes were the independent variables. The results suggested that the high expression levels of hsa_circ_0008507 {(OR = 1.30; 95% CI: 1.13–1.51 in Model 1), (OR = 1.29; 95% CI: 1.11–1.50 in Model 2)}, hsa_circ_0001946 {(OR = 1.25; 95% CI: 1.06–1.47 in Model 1), (OR = 1.20; 95% CI: 1.01–1.42 in Model 2)}, hsa_circ_0000284 {(OR = 1.88; 95% CI: 1.24–2.84 in Model 1), (OR = 2.05; 95% CI: 1.32–3.19 in Model 2)} were the independent risk factors of CHD in the two models (Table 4).

**Table 4:** Multivariable logistic regression analyses of the verified circRNAs and environmental factors in CHD risk

Factors	Model 1		Model 2	
	OR (95% CI)	*p*-value	OR (95% CI)	*p*-value
hsa_circ_0008507	1.30 (1.13-1.51)	< 0.001	1.29 (1.11-1.50)	0.001
hsa_circ_0001946	1.25 (1.06-1.47)	0.007	1.20 (1.01-1.42)	0.033
hsa_circ_0000284	1.88 (1.24-2.84)	0.003	2.05 (1.32-3.19)	0.002
hsa_circ_0125589	0.97 (0.73-1.29)	0.821	0.960 (0.71-1.29)	0.779

Model 1: Adjusted for age, gender, marital status, and education level

Model 2: Adjusted for age, gender, marital status, education level, anxiety, BMI and physical activity

*OR* odds ratio, *CI* confidence interval, *BMI* Body mass index

### Determining diagnostic cut-offs by ROC curve

**Fig. 4:**
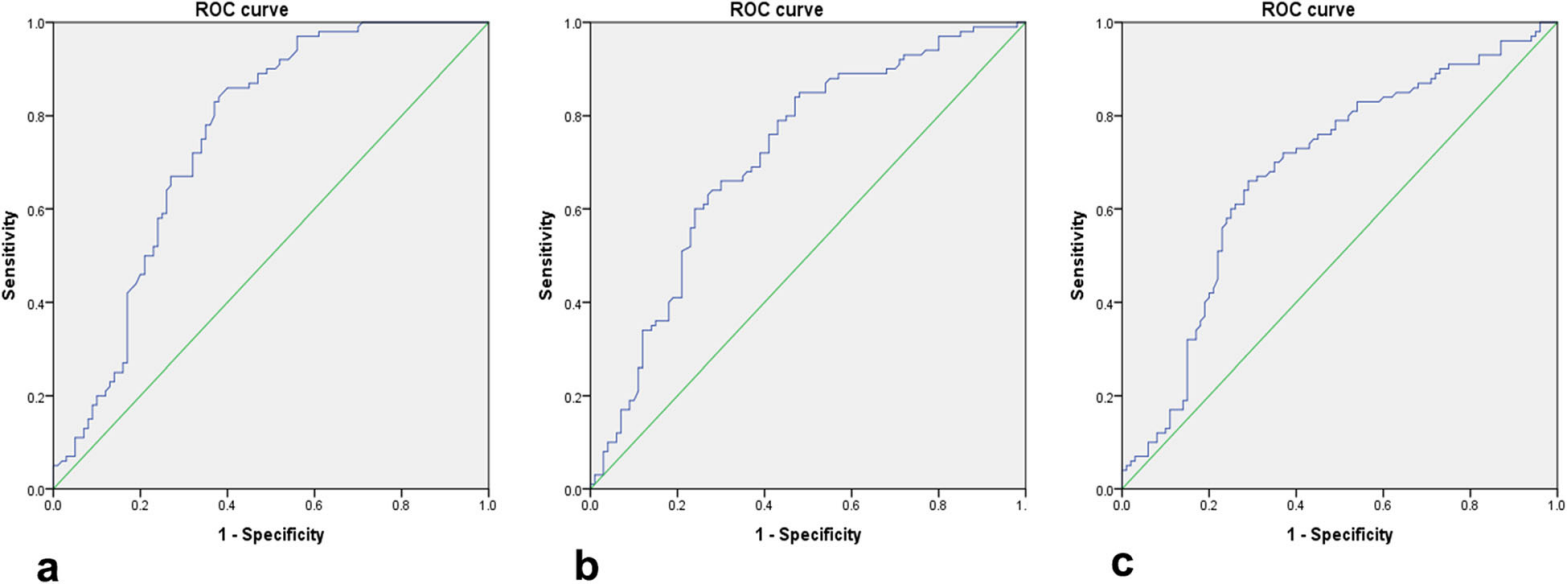
circRNAs in 200 cases of peripheral blood leukocytes in the diagnosis of CHD ROC curve. **a** ROC curve for hsa_circ_0008507; **b** ROC curve for hsa_circ_0001946; **c** ROC curve for hsa_circ_0000284

The ROC curve in 100 cases and 100 controls of peripheral blood leucocytes of hsa_circ_0008507 showed an AUC of 0.75 (95% CI: 0.68–0.82). The maximum Youden’s index was 0.46 with a sensitivity of 0.86 and a specificity of 0.60, corresponding to a diagnostic cut-off of 1.975. The ROC curve of hsa_circ_0001946 showed that the AUC was 0.71 (95% CI: 0.64–0.79). The maximum Youden’s index was 0.37 with a sensitivity of 0.85 and a specificity of 0.52, corresponding to a diagnostic cut-off of 1.00. The ROC curve of hsa_circ_0000284 showed that the AUC was 0.68 (95% CI: 0.61–0.76). The maximum Youden’s index was 0.37 with a sensitivity of 0.66 and a specificity of 0.71, corresponding to a diagnostic cut-off of 1.42 (Fig. 4).

### Combined analysis of environmental factors and epigenetic factors in CHD

The diagnostic cut-offs were used to define high and low expression groups of hsa_circ_0008507, hsa_circ_0001946 and hsa_circ_0000284. Stratification by demographic characteristics and main lifestyle factors revealed the statistically significant associations between hsa_circ_0008507, hsa_circ_0001946 and CHD risk in young and old individuals, males and females, smokers and non-smokers, abdominal obesity and non-abdominal obesity individuals and passive smokers and active smokers. The statistically significant associations between hsa_circ_0000284 and CHD risk were also observed in these comparison groups. In addition, the combined effect of hsa_circ_0008507 and smoking was observed. Compared with non-smoking individuals with low hsa_circ_0008507 expression, smokers with high hsa_circ_0008507 expression showed the highest magnitude of OR in CHD risk (OR 6.94, 95% CI 1.95–24.72). A statistically significant multiplicative interaction was found between hsa_circ_0008507 and smoking for CHD (OR multiplicative: 5.68; 95% CI: 1.08–29.84; *p* = 0.040) (Tables 5 and 6).

**Table 5:** Risk odds ratios of CHD for circRNAs stratified by selected characteristics

	hsa_circ_0008507		hsa_circ_0001946		hsa_circ_0000284	
Variable	Adjusted OR(95% CI)	p_interaction_	Adjusted OR(95% CI)	p_interaction_	Adjusted OR(95% CI)	p_interaction_
Age						
< 65	16.82 (4.15-68.20)	0.808	6.75 (1.86-24.43)	0.865	5.39 (1.91-15.22)	0.920
≥ 65	9.70 (3.46-27.15)		6.54 (2.45-17.47)		7.18 (2.62-19.70)	
Gender						
Male	19.18 (5.65-65.09)	0.065	8.89 (2.90-26.66)	0.371	3.47 (1.35-8.93)	0.169
Female	4.88 (1.74-13.67)		3.48 (1.14-10.61)		11.17 (3.44-36.33)	
Smoking						
No	4.82 (1.96-11.88)	0.040	3.60 (1.35-9.59)	0.070	10.18 (3.66-28.33)	0.148
Yes	25.25 (5.84-109.22)		13.30 (3.50-50.49)		3.12 (1.010-9.63)	
Abdominal obesity						
No	18.34 (2.46-136.77)	0.931	6.58 (1.22-35.61)	0.799	11.65 (1.72-78.94)	0.476
Yes	11.30 (4.31-29.58)		7.93 (2.92-21.54)		4.48 (2.03-10.34)	
Passive smoking						
No	8.78 (3.57-21.61)	0.747	4.80 (2.00-11.49)	0.363	5.17 (2.31-12.49)	0.969
Yes	11.72 (2.07-66.40)		42.52 (3.28-551.24)		3.47 (0.77-15.64)	

Data are adjusted for age, gender, marital status, education level, anxiety, BMI, and physical activity

*OR* odds ratio, *CI* confidence interval

**Table 6:** Joint effect of smoking and hsa_circ_0008507

Smoking	hsa_circ_0008507 high expression	Cases *N* (%)	Controls *N* (%)	OR(95% CI)	*P*-value
0	0	11 (11.0)	30 (30.0)	1.00	
0	1	54 (54.0)	28 (28.0)	4.84 (1.97-11.88)	0.001
1	0	3 (3.0)	30 (30.0)	0.25 (0.05-1.21)	0.085
1	1	32 (32.0)	12 (12.0)	6.94 (1.95-24.72)	0.003
Multiplication interaction				5.68 (1.08-29.84)	0.040

Data are adjusted for age, gender, marital status, education level, anxiety, BMI, and physical activity

## Discussion

We confirmed that high BMI and hsa_circ_0008507, hsa_circ_0001946, hsa_circ_0000284 expression levels are associated with CHD, thereby indicating that CHD is a complex disease affected by multiple levels of factors. Furthermore, combined analysis results show that smoking combined with hsa_circ_0008507 induces the development of CHD.

Among the common risk factors, high BMI is associated with a significantly increased risk of CHD, and this finding is consistent with the report of Fritz J et al. and Y Chen et al. [22, 23]. Increased body mass accompanied by high-powered circulation, chronic volume overload and increased preload and afterload caused by increased peripheral resistance is a risk factor for cardiovascular disease [24].

The expression levels of hsa_circ_0001946, hsa_circ_0008507 and hsa_circ_0000284 were significantly elevated in CHD peripheral blood leukocytes. The overexpression of Hsa_circ_0001946 (also called Cdr1as) in cardiac myocyte cells promotes cell apoptosis [20]. In the present study, hsa_circ_0001946 was up-regulated in CHD, and miR-7-5p was found in its target gene based on bioinformatics analysis. miR-7-5p directly inhibits epidermal growth factor receptor mRNA, further antagonises downstream protein kinases, induces apoptosis and inhibits cell proliferation, migration and invasion [25]. Therefore, we speculate that hsa_circ_0001946 may be involved in the development of vascular endothelium through the hsa_circ_0001946/miR-7-5p pathway.

Hsa_circ_0000284 (also called circHIPK3) mediates retinal vascular dysfunction in diabetes mellitus [21]. Retinal endothelial cell viability and endothelial cell abnormal proliferation, migration and tube formation in vitro and alleviates retinal vascular dysfunction was reduced by silencing of circHIPK3, such as reducing vascular leakage and inflammation, as well as the number of diabetic-induced cellular capillaries in vivo. Population experimental studies showed that hsa_circ_0000284 is associated with carotid plaque rupture and stroke [26]. These results reveal that hsa_circ_0000284 is closely related to the growth, proliferation and plaque rupture of vascular endothelial cells.

The qRT-PCR results were incompletely consistent with those of the microarray analysis. The possible causes for this contradiction are as follows: (1) microarray analysis is only a tool for initial screening. It has high sensitivity and poor reliability. Therefore, the final result is based on the PCR verification result. (2) Differences in research objects: the same sample could not satisfy the requirement for microarray analysis and PCR verification at the same time due to the limited number of experimental samples. Therefore, the two groups of subjects tested on the microarray analysis and validated by PCR are different. (3) Sample size: the microarray analysis is costly; only five cases and five controls were selected, and the individual differences were not excluded.

Peripheral blood leukocytes can participate in the development of CHD through processes, such as blood vessel adhesion [27, 28]. Therefore, large-sample peripheral blood leukocyte qRT-PCR verification was further conducted. The results show that hsa_circ_0125589, hsa_circ_0008507, hsa_circ_0001946 and hsa_circ_0000284 are consistent in peripheral blood and peripheral blood leukocytes.

Stratified analysis shows that hsa_circ_0008507, hsa_circ_0001946 and hsa_circ_0000284 exhibit significant differences in various populations. A statistically significant multiplicative interaction is found between hsa_circ_0008507 and smoking. Smoking can reduce the bioavailability of nitric oxide, further promote the expression of adhesion molecules and endothelial dysfunction and increase the adhesion of platelets and macrophages, thereby leading to coagulation and inflammation. Smoking can also promote the differentiation of macrophages into foam cells, thereby aggravating the progression of CHD [29, 30]. Therefore, the interaction between smoking and the high expression of hsa_circ_0008507 results in a high risk of CHD.

This study has the following deficiencies. (1) In this case–control study, we collected the subject’s exposure through a structured questionnaire. Although objective data were selected as much as possible, the possibility that the respondent’s memory is distorted or incomplete still exists. Hence, information bias is inevitable. (2) The representative sample is insufficient because the cases and controls were only obtained from two hospitals in Fuzhou, indicating admission rate bias. (3) The expression level of circRNAs cannot be dynamically evaluated due to the lack of detection at multiple time points and the limitation on the causal association between circRNA and CHD.

## Conclusion

Hsa_circ_0008507, hsa_circ_0001946 and hsa_circ_0000284 are closely related to the occurrence and development of CHD. In addition, the combination of smoking and high hsa_circ_0008507 expression induces the occurrence and development of CHD.

## Additional files

Additional file 1: **Table S1.** the questionnaire used for investigation. The questionnaire included demographic characteristics (age, gender, marital status, educational level), lifestyle habits (smoking, alcohol drinking, diet, exercise), social psychological factors (, anxiety and depression levels), Physiological index (height, weight, waist circumference (WC)) and family history of cardiovascular disease. (DOC 62 kb)

Additional file 2: **Table S2.** Baseline characteristics of subjects used for microarray analysis. Five patients with similar age, disease and disease duration and with no other diseases and five controls with similar general conditions, age and sex was selected for microarray analysis. (DOC 37 kb)

Additional file 3: **Table S3.** List of the primers used for qRT-PCR experiments. The primers used for qRT-PCR experiments was listed in **Table S3**. (DOC 32 kb)

## Data Availability

The datasets generated and/or analysed during the current study are not publicly available due the principle of confidentiality of funding but are available from the corresponding author on reasonable request.

## Abbreviations

AUC: Area under the curve
BMI: Body mass index
CHD: Coronary heart disease
CI: Confidence interval
CircRNAs: Circular RNAs
OR: Odds ratio
qRT-PCR: Quantitative real-time polymerase chain reaction
ROC: Receiver-operating characteristic
