# Supplementary material for: Association of circular RNAs and environmental risk factors with coronary heart disease
Source: BMC Cardiovasc Disord. 2019 Oct 16;19:223. doi: 10.1186/s12872-019-1191-3 (PMC6796436; doi:10.1186/s12872-019-1191-3)
Supplement: Supplementary file 1 — Table S1. the questionnaire used for investigation. The questionnaire included demographic characteristics (age, gender, marital status, educational level), lifestyle habits (smoking, alcohol drinking, diet, exercise), social psychological factors (, anxiety and depression levels), Physiological index (height, weight, waist circumference (WC)) and family history of cardiovascular disease. (DOC 62 kb) [file 12872_2019_1191_MOESM1_ESM.doc]

**Questionnaire**

Survey signature：

Date：

一、**General demographic characteristics**

1. **Gender：**①Man ②Woman
2. **Age：**­________
3. **Marital status：**①Unmarried ②Married ③Widowed ④Divorced ⑤Separation

**4. Family history：**Hypertension【①Yes ②No】Diabetes【①Yes ②No】stroke【①Yes ②No】

**二、Lifestyle habits**

1.Height: cm;Weight: kg;Waistline: cm;Hip circumference:­­______cm.

2.Education level: ①Below primary school ②Middle school ③College or higher

3.Smoking ? ①No ②Yes

4.Passive smoking ? ①Yws ②No

5.Alcohol drinking ? ①No ②Yes

6.High-salt diets ? ①No ②Yes

7. Light diets ? ①No ②Yes

8.Physical exercise ? ①< 1 time/week ② 1-2 times/week ③ ≥3 times/week

9.Character type ? ①Type A ②Type B ③Type C ④Type D

**三、Clinical diagnosis**

1. Coronary heart disease：①No ②Yes

**四、SAS and SDS**

| Item | No or almost no | a small part of the time | Quite a lot of time | Almost always  Almost always |
| --- | --- | --- | --- | --- |
| 1.I feel unhappy and depressed. | 1 | 2 | 3 | 4 |
| 2.I think the morning is the best in the day. | 1 | 2 | 3 | 4 |
| 3.I burst into tears or wanted to cry. | 1 | 2 | 3 | 4 |
| 4.I don't sleep well at night. | 1 | 2 | 3 | 4 |
| 5.I eat as much as usual. | 1 | 2 | 3 | 4 |
| 6.When I was in close contact with the opposite sex, I felt as happy as ever. | 1 | 2 | 3 | 4 |
| 7.I noticed that my weight is falling. | 1 | 2 | 3 | 4 |
| 8.I have the trouble of constipation. | 1 | 2 | 3 | 4 |
| 9.My heart beats faster than usual. | 1 | 2 | 3 | 4 |
| 10.I feel tired for no reason. | 1 | 2 | 3 | 4 |
| 11.My mind is as clear as usual. | 1 | 2 | 3 | 4 |
| 12.I don't think there are any difficulties in doing things that are often done. | 1 | 2 | 3 | 4 |
| 13.I feel uneasy and can't calm down. | 1 | 2 | 3 | 4 |
| 14.I have hope for the future. | 1 | 2 | 3 | 4 |
| 15.I am more excited than usual. | 1 | 2 | 3 | 4 |
| 16.I think it is easy to make a decision. | 1 | 2 | 3 | 4 |
| 17.I feel that I am a useful person and someone needs me. | 1 | 2 | 3 | 4 |
| 18.My life has been very interesting. | 1 | 2 | 3 | 4 |
| 19.I think that if I don't exist, others will live better. | 1 | 2 | 3 | 4 |
| 20.I am still interested in things that are usually of interest. | 1 | 2 | 3 | 4 |
| 21.I feel more nervous or anxious than usual. | 1 | 2 | 3 | 4 |
| 22.I am afraid for no reason. | 1 | 2 | 3 | 4 |
| 23.I am easily upset or frightened. | 1 | 2 | 3 | 4 |
| 24.I think I may be going crazy. | 1 | 2 | 3 | 4 |
| 25.I think everything is fine and there will be no misfortune. | 1 | 2 | 3 | 4 |
| 26.My hands and feet are shaking and shaking. | 1 | 2 | 3 | 4 |
| 27.I am troubled by headaches, neck pains and back pains. | 1 | 2 | 3 | 4 |
| 28.I feel prone to weakness or fatigue. | 1 | 2 | 3 | 4 |
| 29.I feel calm and easy to sit quietly. | 1 | 2 | 3 | 4 |
| 30.I feel that my heart beats very quickly. | 1 | 2 | 3 | 4 |
| 31.I am distressed by the dizziness. | 1 | 2 | 3 | 4 |
| 32.I have fainted or felt like I was fainting. | 1 | 2 | 3 | 4 |
| 33.I feel very easy to breathe in and exhale. | 1 | 2 | 3 | 4 |
| 34.My hands and feet are numb and stinging. | 1 | 2 | 3 | 4 |
| 35.I am troubled by stomach pain and indigestion. | 1 | 2 | 3 | 4 |
| 36.I often have to urinate. | 1 | 2 | 3 | 4 |
| 37.My hands and feet are often dry and warm. | 1 | 2 | 3 | 4 |
| 38.I am blushing. | 1 | 2 | 3 | 4 |
| 39.I am easy to fall asleep and sleep well overnight. | 1 | 2 | 3 | 4 |
| 40.I always have nightmares. | 1 | 2 | 3 | 4 |

Investigator： Date of investigation： Quality controller： Date of review：
