# Supplementary material for: Association of circular RNAs and environmental risk factors with coronary heart disease
Source: BMC Cardiovasc Disord. 2019 Oct 16;19:223. doi: 10.1186/s12872-019-1191-3 (PMC6796436; doi:10.1186/s12872-019-1191-3)
Supplement: Supplementary file 2 — Table S2. Baseline characteristics of subjects used for microarray analysis. Five patients with similar age, disease and disease duration and with no other diseases and five controls with similar general conditions, age and sex was selected for microarray analysis. (DOC 37 kb) [file 12872_2019_1191_MOESM2_ESM.doc]

**Table S2** Baseline characteristics of subjects used for microarray analysis

| Characteristics | CHD, n(%)/X±S | Non-CHD, n(%)/X±S | χ^2^/T | p-value |
| --- | --- | --- | --- | --- |
| Gender |  |  | - | 1.000^a^ |
| Male | 3(60.0) | 3(60.0) |  |  |
| Female | 2(40.0) | 2(40.0) |
| Age^b^ | 67.00±8.22 | 59.40±6.23 | -1.648 | 0.138 |
| Marital status |  |  | - | - |
| Marriage | 5(100.0) | 5(100.0) |  |  |
| Single and others | 0(0.0) | 0(0.0) |
| Education level |  |  | - | 1.000^a^ |
| Below primary school | 4(80.0) | 4(80.0) |  |  |
| Middle school | 1(20.0) | 1(20.0) |  |  |
| Smoking |  |  | - | 1.000^a^ |
| No | 4(80.0) | 3(60.0) |  |  |
| Yes | 1(20.0) | 2(40.0) |  |  |
| Alcohol drinking |  |  | - | 1.000^a^ |
| No | 1(20.0) | 0(0.0) |  |  |
| Yes | 4(80.0) | 5(100.0) |  |  |
| Waist circumference^b^ | 94.80±11.12 | 85.13±24.88 | -0.787 | 0.457 |
| BMI^b^ | 26.03±4.04 | 24.73±3.51 | -0.543 | 0.602 |

a:Fisher’s Exact Test

b:Two-tailed Student' s t-test
