# Supplementary material for: Association of circular RNAs and environmental risk factors with coronary heart disease
Source: BMC Cardiovasc Disord. 2019 Oct 16;19:223. doi: 10.1186/s12872-019-1191-3 (PMC6796436; doi:10.1186/s12872-019-1191-3)
Supplement: Supplementary file 3 — Table S3. List of the primers used for qRT-PCR experiments. The primers used for qRT-PCR experiments was listed in Table S3. (DOC 32 kb) [file 12872_2019_1191_MOESM3_ESM.doc]

| **Table S3** List of the primers used for qRT-PCR experiments | | |
| --- | --- | --- |
| Name | Primer | Sequence（5’-3’） |
| GAPDH | Forward | GCACCGTCAAGGCTGAGAAC |
|  | Reverse | TGGTGAAGACGCCAGTGGA |
| hsa_circ_0125589 | Forward | GGCCTGCAGGATTTGGTTTCA |
|  | Reverse | ATGGTCTGGCTGCGTTGTTG |
| hsa_circ_0008507 | Forward | CGAAGCAATATTGGCTGCCCT |
|  | Reverse | TCGCCCACAACAACACACTTA |
| hsa_circ_0001946 | Forward | AGTCTTCCATCAACTGGCTCA |
|  | Reverse | GACACAGGTGCCATCGGA |
| hsa_circ_0000284 | Forward | TATGTTGGTGGATCCTGTTCGGCA |
|  | Reverse | TGGTGGGTAGACCAAGACTTGTGA |
